# Based on CiteSpace Insights into *Illicium verum* Hook. f. Current Hotspots and Emerging Trends and China Resources Distribution

**DOI:** 10.3390/foods13101510

**Published:** 2024-05-13

**Authors:** Zhoujian He, Jie Huan, Meng Ye, Dan Liang, Yongfei Wu, Wenjun Li, Xiao Gong, Liqiong Jiang

**Affiliations:** 1 College of Forestry, Sichuan Agricultural University, Huimin Road 211, Wenjiang District, Chengdu 611130, China; hezhouj@163.com (Z.H.); gongxiao202209@163.com (X.G.); 2 School of Life Sciences, Sichuan University, Wangjiang Road 29, Wuhou District, Chengdu 610064, China; 3 Enyang District Agriculture and Rural Bureau of Bazhong City, No. 6, Planning Road 40, Enyang District, Bazhong 636600, China; 15388135911@163.com; 4 Baoxing County Natural Resources and Planning Bureau of Yaan City, Lingxiu Road 256, Baoxing County, Yaan 625700, China; 2020206034@stu.sicau.edu.cn; 5 Animal Nutrition Institute, Sichuan Agricultural University, Huimin Road 211, Wenjiang District, Chengdu 611130, China; wyf061003@163.com; 6 Institute of Forestry, Chengdu Academy of Agriculture and Forestry Sciences, Nongke Road 200, Wenjiang District, Chengdu 611130, China; lwj13980429970@163.com (W.L.); liqiong_jiang@163.com (L.J.)

**Keywords:** *Illicium verum*, resource, CiteSpace, bibliometrics

## Abstract

*Illicium verum* Hook. f. is a globally significant spice, which is recognized in China as a food-medicine homolog and extensively utilized across the pharmaceutical, food, and spice industries. China boasts the world’s leading resources of *I. verum*, yet its comprehensive utilization remains relatively underexplored. Through a resource survey of *I. verum* and the application of bibliometric visualization using CiteSpace, this study analyzed 324 papers published in the Web of Science Core Collection (WOSCC) from 1962 to 2023 and 353 core documents from China’s three major databases (CNKI, Wanfang Database, and VIP Database). *I. verum* from Guangxi province towards various southern provinces in China, with autumn fruits exhibited superior quality and market value over their spring fruits. Literature in WOSCC emerged earlier, with a research emphasis on food science technology and pharmacology pharmacy domains. WOSCC research on *I. verum* could be divided into two phases: an embryonic period (1962–2001) and a growth period (2002–2023), showing an overall upward trend in publication. The three major Chinese databases contain a larger number of publications, with a focus on the food sector, which could be categorized into three stages: an embryonic period (1990–1999), a growth period (2000–2010), and a stable period (2011–2023), with an overall downward trend in publication. Both Chinese and international research hotspots converge on the medical applications of *I. verum*, with antioxidant bioactivity research emerging as a prevailing trend. This study delineated the resource distribution of *I. verum* across China and identified the research hotspots and trends both in China and internationally. The findings are beneficial for guiding researchers in swiftly establishing their research focus and furnishing decision-makers with a comprehensive reference for industry information.

## 1. Introduction

Star anise (*Illicium verum* Hook. f.), as known globally, generates an annual output value exceeding 10 billion RMB [1] and is extensively utilized in both the food and pharmaceutical industries [2]. This spice is a common ingredient in the culinary traditions of China and Southeast Asia, enhancing the flavor of meat dishes [3], and it is also employed in Western countries for the production of sweet wines, confections, and cakes [4]. Beyond its culinary applications, *I. verum* is recognized as a medicinal resource, possessing antimicrobial, antiviral, and anti-inflammatory properties [5]. It features in traditional Chinese medicine and the Indian healthcare system [4]. The plant is rich in bioactive compounds such as shikimic acid, essential oil, tannins, and anethole (85–90%), which can inhibit various viruses, including the avian influenza virus H5N1 and the human influenza virus H1N1 [6,7]. These compounds also find their way into daily chemical products [1]. In China, *I. verum* is classified as a food-medicinal plant and has been included in successive editions of the Chinese Pharmacopoeia [8]. The *I. verum* belongs to the Schisandraceae Blume family, within the *Illicium* L. genus [9], and is widely distributed across the southwestern part of the Asian continent [4], with the primary production areas located in the subtropical provinces of southern China [1]. China has a history of cultivating *I. verum* spanning over 300 years [10] and is the sole large-scale exporter of *I. verum* globally [11]. Understanding the distribution of this valuable resource is crucial for its optimal development and utilization. *I. verum* holds significant economic value on a global scale [6,7,8]. Gaining insights into its research hotspots and trends is anticipated to further stimulate the development and application of this valuable spice. However, the current landscape is replete with a vast body of literature on *I. verum*, both within China and internationally. Traditional literature review methods are often insufficient to extract the hotspot and trend information pertinent to *I. verum* research, underscoring the need for advanced analytical tools to navigate this complex field.

Literature review is instrumental in understanding the cutting-edge developments within a field. However, the sheer volume of publications in databases presents a challenge in achieving a comprehensive grasp of the domain’s evolution [12,13]. Traditional reviews often focus on a specific domain, and it is seldom feasible to encompass the entirety of the literature to analyze the current state of research within a particular field [13], thus necessitating more convenient methods for literature analysis. Rapidly identifying the developmental hotspots and trends within a field can significantly save researchers time by swiftly guiding them toward promising research directions [10,14]. Moreover, such insights also serve to provide a more comprehensive industry information base, which is invaluable for strategic planning and decision-making [15,16]. Bibliometrics offers a solution by visualizing the literature, which can be employed to explore the dynamic changes in past research and predict future trends [14,15]. CiteSpace is a pivotal statistical software within the realm of bibliometrics, capable of rapidly mapping subject networks and assisting researchers in identifying trends and hotspots [16,17,18]. The application of the CiteSpace tool has facilitated the identification of research hotspots and trends across a spectrum of scientific disciplines, thereby significantly advancing scientific inquiry. Notably, this tool has been instrumental in fields such as food science [12,19], medicine [20], traditional Chinese medicine [21], and ecology [22], where it has helped to distill complex information and highlight areas ripe for further exploration. *I. verum*, as a globally significant research material, has been the subject of several literature reviews. However, these reviews often focus on food or medical aspects [2,23] and do not adequately reflect the dynamic evolution of research in this area.

The present study synthesizes the distribution of *I. verum* resources in China and employs CiteSpace to visualize journal articles from the three major Chinese databases and the Web of Science Core Collection (WOSCC). By doing so, we analyze the trends and hotspots in both Chinese and international research on *I. verum*. The findings of this study not only showcase the current state of *I. verum* resources in China but also serve as a reference for policymakers and growers. Furthermore, this research facilitates a better understanding for scientific researchers of the dynamic evolution and emerging trends within *I. verum* studies.

## 2. Materials and Methods

International literature databases such as Scopus and Google Scholar are available, but the Web of Science (WOS) is more suited for large-scale bibliometric analysis [24]. The WOSCC is an integral component of the WOS database, encompassing a wide array of influential literature from around the globe. The subject search was conducted using the search function of WOSCC, employing the following strategy: Topical Subject = Illicium verum OR Title = Illicium verum OR Author Keywords = Illicium verum OR Abstract = Illicium verum; the language was restricted to English, and the document type was limited to articles. The search was accessed on 25 March 2024. Literature published in 2024 was excluded, and the analysis focused on articles, resulting in a selection of 324 papers for analysis.

**Figure 1:**
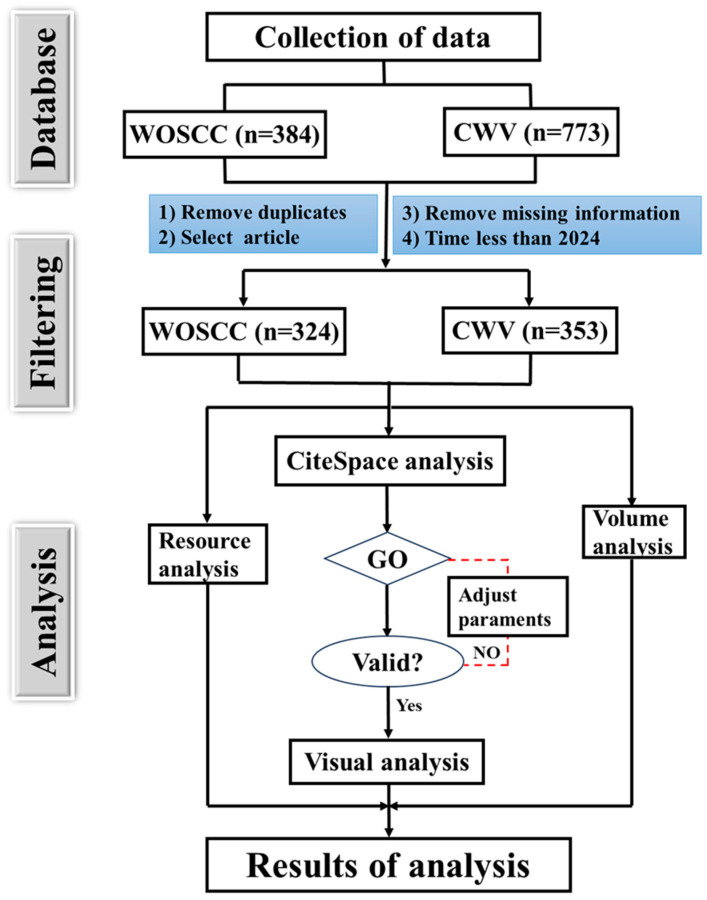
The process of literature analysis. CWV: CNKI (https://www.cnki.net/), Wanfan data (https://w.wanfangdata.com.cn/index.html?index=true), and VIP data (http://www.cqvip.com/) about publications of *I. verum*. WOSCC: Web of Science core collection about publications of *I. verum*.

The literature search was conducted across three Chinese databases: China National Knowledge Infrastructure (CNKI https://www.cnki.net/, accessed on 8 February 2024), Weipu Database (VIP http://www.cqvip.com/, accessed on 8 February 2024), and the Wanfang Database (https://w.wanfangdata.com.cn/index.html?index=true, accessed on 8 February 2024). The search criteria included publications with “Illicium verum” in the subject or title from 1990 to 2023. The query was restricted to journals indexed as core by Peking University, and duplicates were removed. This process yielded a total of 353 papers for analysis. The specific analytical procedure is depicted in Figure 1.

Visualization of the literature was performed using CiteSpace V.6.2.R4 (Drexel University, Philadelphia, PA, USA), with the following parameter settings: (1) Time slicing was set from 1962 to 2023, with a slice width of 5 years; (2) The g-index was configured with a k-value of 25; (3) For network pruning, the Pathfinder and slicing network pruning algorithms were selected. Keyword co-occurrence maps were generated using the Log-Likelihood Ratio (LLR) algorithm to extract keywords. All clustering results had modularity above 0.3 and silhouette values above 0.7, indicating robust clustering outcomes [12].

## 3. Results

### 3.1. Resource of I. verum

**Figure 2:**
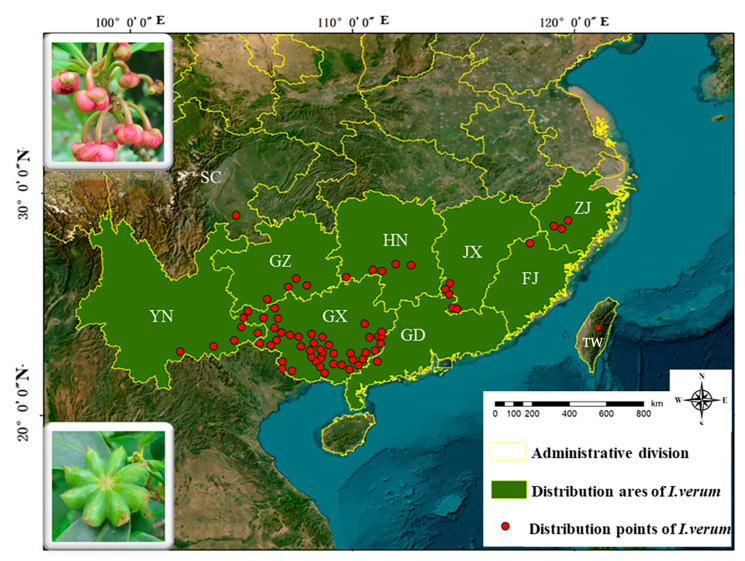
Distribution of *I. verum* in China. The green-shaded regions in the figure delineate the primary areas of *I. verum* cultivation in China, while the red dots indicate the distribution points of *I. verum* across various provinces. YN: Yunnan province; SC: Sichuan province; GZ: Guizhou province; GX: Guangxi Zhuang Autonomous Region; HN: Hunan province; GD: Guangdong province; JX: Jiangxi province; FJ: Fujian province; ZJ: Zhejiang province; TW: Taiwan province. Morphological images of *I. verum* fruits and flowers. The image was created using ArcGIS 10.5.

In this study, we have mapped the primary distribution areas of the *I. verum* resources in China [1,9,25,26,27,28], as depicted in Figure 2. According to the literature, *I. verum* was predominantly found in the southern regions of the country [1], near the tropic of cancer [11]. The resource was mainly distributed across provinces such as Guangxi, Yunnan, Guangdong, Guizhou, Hunan, Jiangxi, Fujian, and Zhejiang, with small-scale cultivation in the Shigang Forest Farm in Lu County, Sichuan, and sporadic plantings in other provinces of the south [1]. Among the cultivating provinces, Guangxi had the largest planting area, covering 36,506 km^2^ across 24 counties (cities or districts), with Baise and Ningming counties known for their superior quality and cultivation history exceeding 300 years [1,28]. Longzhou County, Fangcheng District, and Teng County are the top producers of *I. verum*, with an output exceeding 200,000 kg. The distribution of *I. verum* resources was centered in Guangxi and radiated to the adjacent southern provinces.

In the *Illicium*, certain species closely resemble *I. verum*, leading to the emergence of counterfeit *I. verum* that was challenging to distinguish from the authentic product. These counterfeits had not only disrupted the market for *I. verum* but had been responsible for incidents of poisoning due to their toxic properties. Here, we listed the common adulterants of *I. verum* and their toxic parts, as depicted in Table 1.

**Figure 3:**
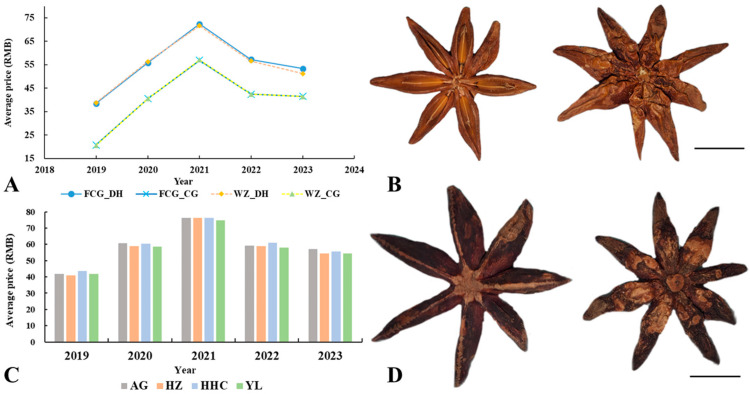
Price and Morphology of Two *I. verum* Varieties from 2019 to 2023. (**A**) Price of *I. verum* in Fangchenggang and Wuzhou cities in the Guangxi Zhuang Autonomous Region. FCG_CG: spring fruits of *I. verum* in Fangchenggang city; FCG_DH: autumn fruits of *I. verum* in Fangchenggang city; WZ_CG: spring fruits of *I. verum* in Wuzhou city; WZ_DH: autumn fruits of *I. verum* in Wuzhou city. (**C**) Annual price changes for autumn fruits of *I. verum* in four major traditional Chinese medicine markets in China. AG: Anguo Medicine Market in Baoding; HZ: Heze Medicine Market in Bozhou; HHC: Hehuachi Medicine Market; YL: Yulin Medicine Market in Yulin. (**B**) Morphological features of autumn fruits of *I. verum*. (**D**) Morphological features of spring fruits of *I. verum*. The prices depicted in the figure represent the average annual values for each variety. Scale = 1 cm.

In addition to the presence of morphologically similar counterfeits, *I. verum* was often classified into two varieties based on the harvest time: the spring fruit (horn flower star anise) and the autumn fruit (big red star anise). These two varieties differ not only in their chemical composition but also in their market value [29]. Data from the Chinese Medicinal Materials World Network (https://www.zyctd.com/jiage/xq8.html, accessed on 6 March 2024), which span from 2019 to 2023, provides a detailed analysis of the prices of different types of *I. verum* across various trading markets, as well as the morphological characteristics of the spring and autumn fruits, as depicted in Figure 3. The price trend of *I. verum* exhibited an overall “inverted V” pattern, reaching its peak in 2021 before stabilizing. Despite this stabilization, prices remained higher compared to those in 2019 and 2020. Interestingly, the price of the spring fruit is consistently lower than that of the autumn fruit across different regions, with minimal price fluctuations observed within the same type of fruit (Figure 3A). Within the same year, the price of autumn fruit was relatively constant across different trading markets (Figure 3C). Morphologically, the autumn fruit of *I. verum* was characterized by a better opening degree and a bright yellow fruit color, which gives it an overall superior appearance compared to the spring fruit (Figure 3B,D). The microscopic morphology of *I. verum* is illustrated in Figure S1. These findings underscore the importance of distinguishing between the different varieties of *I. verum* not only to combat counterfeit products but also to understand the economic implications of seasonal variations in the harvest.

### 3.2. Annual Number and Trend of Publications

**Figure 4:**
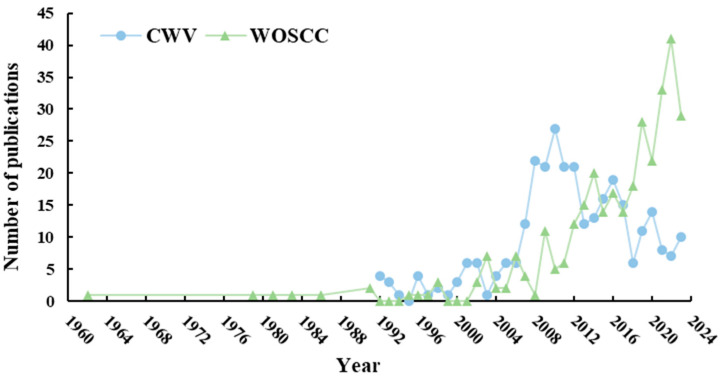
Trends of the number of publications issued by *I. verum*. CWV: CNKI (https://www.cnki.net/), Wanfan data (https://w.wanfangdata.com.cn/index.html?index=true), and VIP data (http://www.cqvip.com/) about publications of *I. verum*. WOSCC: Web of Science core collection about publications of *I. verum*.

The annual variation in the number of publications was a significant metric for gauging the academic research activity within a field [30], and it could reflect the pace of research development [31]. An analysis of articles related to *I. verum* in three Chinese databases and the WOSCC was presented in Figure 4. The initial publication of literature in WOSCC occurred in 1962, predating the first literature in three Chinese databases, which appeared in 1990. The annual publication trend of literature in three Chinese databases could be divided into three distinct phases: the embryonic phase (1990–1999), during which the number of publications was relatively low; the growth phase (2000–2010), characterized by an overall upward trend in publication numbers, peaking at 29 articles in 2010; and the stable phase (2011–2023), where the overall publication trend experienced a decline, yet the number of publications remained above seven per year. These findings provided valuable insights into the trajectory of academic interest and research output in the field of *I. verum*, highlighting the dynamic nature of research focus and the potential for future exploration.

The growth trend of literature in WOSCC on *I. verum* could be delineated into two distinct phases. The first phase, termed the embryonic phase (1962–2001), was characterized by an unstable number of publications. The second phase, known as the growth phase (2002–2023), witnessed an overall upward trend in publication volume, culminating in a peak of 41 articles in the year 2022.

### 3.3. Main Research Forces

#### 3.3.1. Countries and Institutions

**Figure 5:**
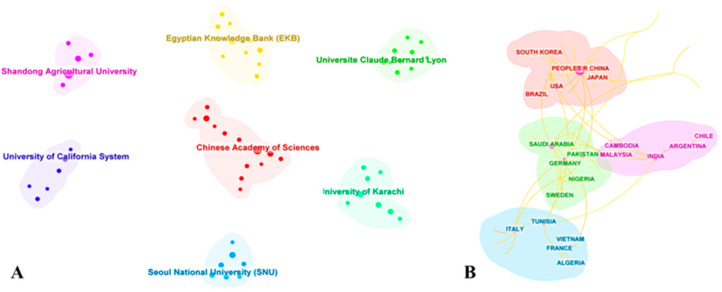
Network visualization map of cooperation between countries and institutions. (**A**) CiteSpace network map of institutions involved in *I. verum*, Modularity (Q = 0.9243), Silhouette (S = 0.9903). (**B**) CiteSpace network map of countries involved in *I. verum*, the size of the circle indicates the number of countries that had published papers, and the yellow line indicates the cooperation between different countries. Only the top 20 countries with the number of articles were shown. Modularity (Q = 0.5526), Silhouette (S = 0.7253).

International scientific collaboration played a significant role in integrating into the global research community, reflecting the collaborative relationships between different countries and academic institutions [32]. The WOSCC, as a pivotal bibliographic database, effectively mirrors international cooperation. By employing CiteSpace for visualization, we depicted the leading institutions and countries in terms of publication output on *I. verum* within the WOSCC, as shown in Figure 5. Both the Chinese Academy of Sciences and Shandong Agricultural University have published over 10 articles related to *I. verum* (Figure 5A), with all these publications originating from China, indicating a close engagement of Chinese institutions in the research of *I. verum*. Among three Chinese database research institutions, those from Guangxi Zhuang Autonomous Prefecture had the highest number of publications on *I. verum* (Figure S2). The research on *I. verum* spanned over 50 countries and regions, demonstrating its global attention. The country with the most extensive research on *I. verum* was the People’s Republic of China, with over 120 publications (Figure 5B). The size of the circles was proportional to the number of publications from each country [33]. Among the top 10 countries in *I. verum* research, the majority were located in Asia. In summary, the visualization analysis of research institutions and countries indicated a close correlation between the volume of scientific literature and the distribution of *I. verum* resources.

The tier of research journals serves as a critical instrument for gauging the quality of research [13]. Subsequently, we conducted a statistical analysis of the top 10 journals by publication count, both Chinese and internationally, with the results presented in Table 2. Based on the data from WOSCC, the research field of *I. verum* abroad was primarily concentrated in “Food Science & Technology” (60 publications) and “Pharmacology & Pharmacy” (47 publications). Among the top 10 journals with the highest publication count on *I. verum* in the WOSCC, “Molecules” (11 publications) published the most papers, showcasing a diverse range of research topics. The proportion of journals with a Journal Citation Report (JCR) rank of Q1 is 60%, indicating a high level of research quality. In the three Chinese databases, the research field of *I. verum* was predominantly focused on “Light Industrial Handicrafts” and “Traditional Chinese Medicine”, with a relatively stable research domain. In the top 10 Chinese journals by publication count, “China Condiment” (60 publications) published the most papers. Only the journal “Food Science” was recognized as an outstanding Chinese journal, suggesting that the overall level of research on *I. verum* in Chinese journals was relatively low, and the influence was weak.

#### 3.3.2. Main Author

**Figure 6:**
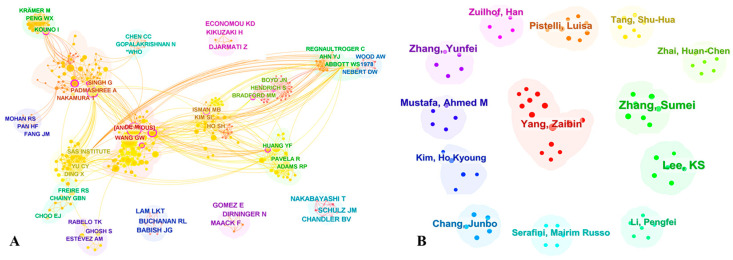
Citespace network of co-cited authorship and TOP 13 authors. (**A**) co-cited authorship in the field of *I. verum* research. The size of the circle is positively correlated with the cited counts of the authors, and the yellow lines represent a collaboration between two authors. Modularity (Q = 0.7921), Silhouette (S = 0.8684). (**B**) TOP 13 author in the field of *I. verum* research. The small number of points within the module reflects the number of articles published by the author. Modularity (Q = 0.9661), Silhouette (S = 0.9828).

The co-citation network of literature represents the structure and development of a field, and identifying key researchers within this network could aid in discovering leading research teams [30,34]. We analyzed the primary authors and co-cited authors within the WOSCC, as depicted in Figure 6. The correlation between publication count and citation frequency in *I. verum* research was not significant. Both the most prolific author and the author with the highest citation count were scholars from China, indicating a deep level of research engagement in this field within the country. Yang Zaibin had the highest number of publications (12 papers), suggesting extensive research contributions. Wang GW had the highest number of citations (114), indicating a high quality of published work. Further analysis of *I. verum* researchers in China’s three major databases was presented in Figure S3. The principal investigator in Chinese *I. verum* research is “Miu Jianhua”, who was characterized by close collaborations with other researchers within China.

### 3.4. Research Trend and Hotspot Analysis

#### 3.4.1. Hotspot in *I. verum*

**Figure 7:**
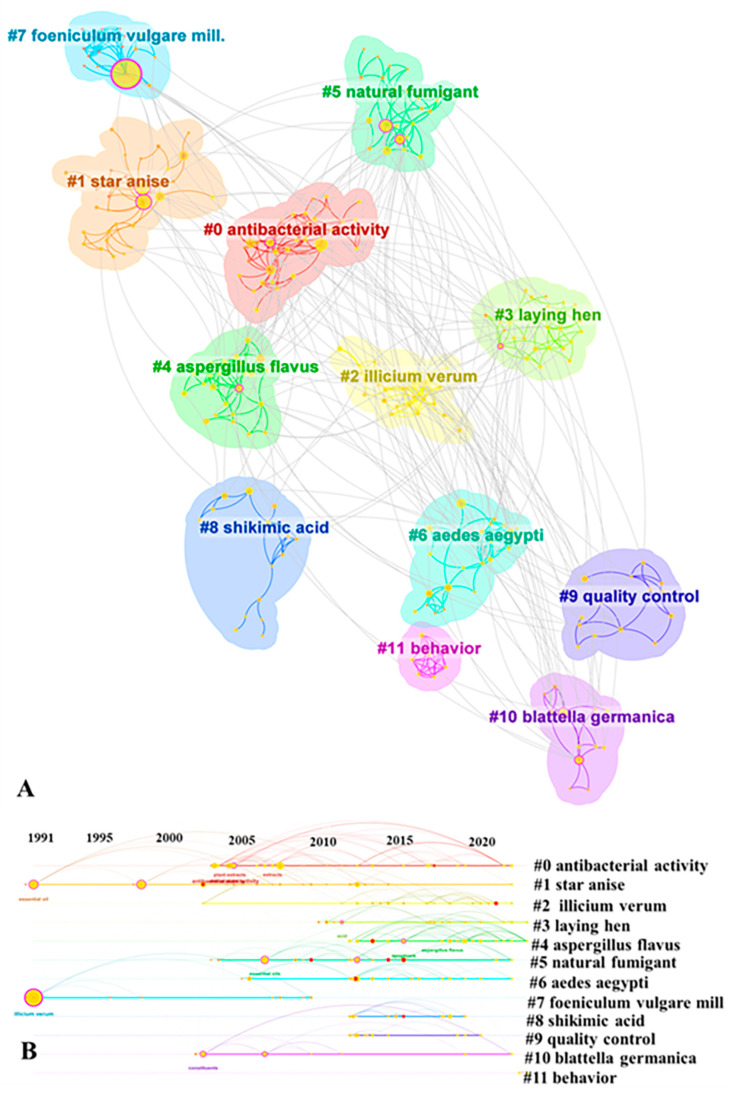
Keyword analysis in *I. verum*-related study. (**A**) Visual mapping of keywords in *I. verum* using CiteSpace; The black lines showed the connections between them. (**B**) The keyword time zones of *I. verum*. Modularity (Q = 0.6483), Silhouette (S = 0.8486).

Understanding the relevance of other related topics could provide insights into a specific field of study [18]. The evolution of article keywords could reflect the structure and dynamics of a knowledge domain [35]. By utilizing CiteSpace to visualize the high-frequency keywords in the *I. verum* literature from the WOSCC, we could reveal the hotspots of research in this field, as shown in Figure 7. The effective clustering results encompass a total of 12 clusters (Figure 7A), which were primarily divided into two main categories. The first category pertains to the classification research of *I. verum* resources, including cluster #1, “star anise”, cluster #2, “Illicium verum”, and cluster #7, “foeniculum vulgare mill”. The timelines of these clusters were notably extensive (Figure 7B), indicating that resource-related issues had become a significant foundation for research on *I. verum*.

The second category pertains to the application research of *I. verum*, which includes cluster #0, “antibacterial activity”, cluster #3, “laying hen”, cluster #4, “Aspergillus flavus”, cluster #5, “natural fumigant”, cluster #6, “Aedes aegypti”, cluster #8, “shikimic acid”, cluster #9, “quality control”, cluster #10, “Blattella germanica”, and cluster #11, “behavior”. The clusters within the application domain of *I. verum* demonstrated a primary focus on the medical and pharmaceutical fields. The keyword clustering of *I. verum* in three Chinese databases (Figures S4 and S5) could likewise be divided into two main categories. However, the three Chinese databases’ keyword clusters were more concentrated on the resources of *I. verum* rather than its applications. Both international and Chinese research on the application of *I. verum* emphasizes the medical field.

#### 3.4.2. Trend in *I. verum*

**Figure 8:**
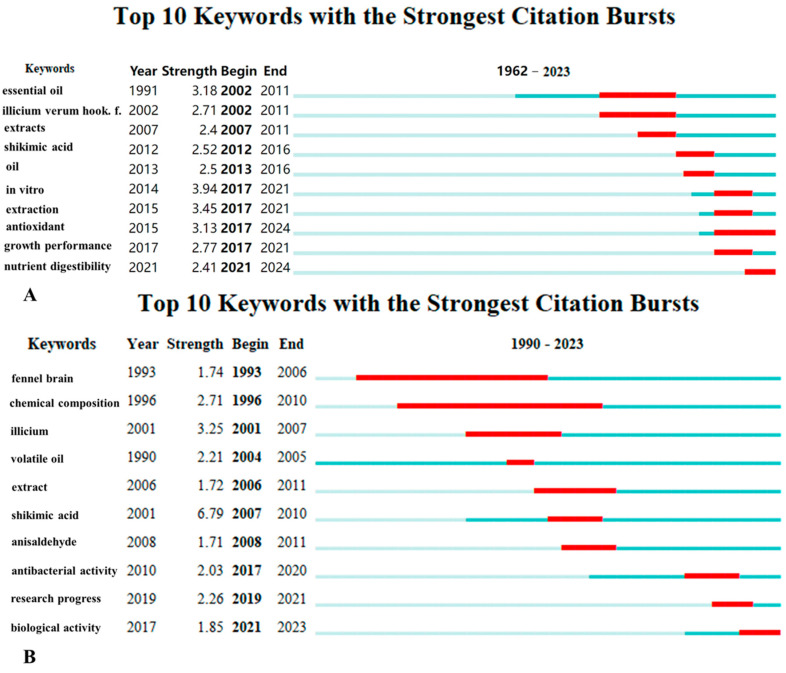
Keywords with the strongest citation bursts of *I. verum*. (**A**) Keywords from 1962 to 2023 appear in WOSCC. (**B**) The keywords of 1990~2023 in the three major Chinese databases appear. The red part indicated that the keyword was in a high-frequency state during the time period.

Keyword clustering analysis enables the identification of emerging research trends [17]. By conducting an analysis of the strongest citation bursts for keywords in both Chinese and international databases, the results are depicted in Figure 8. Based on the top 10 keywords bursts from the WOSCC, the outcomes could be categorized into two groups (Figure 8A): the first group pertains to *I. verum* resources (10%), and the second to the application of *I. verum* (90%), indicating that research on *I. verum* was predominantly focused on its applications. Within the domain of *I. verum* applications, high-frequency keywords could be divided into two categories: the first category involves the chemical composition and extraction methods (60%), and the second category concerns the application of bioactive substances (30%). “Essential oil” and “Illicium verum Hook f” emerged as the earliest high-frequency keywords, suggesting that the initial research on *I. verum* centered on its chemical constituents and resources. While the chemical composition and extraction methods were once the primary high-frequency terms associated with *I. verum*, more recent research trends have shifted towards “antioxidant” and “nutrient digestibility”. The emergence of “nutrient digestibility” as a new high-frequency keyword likely extends from the plant’s antimicrobial and antioxidant properties.

Based on the top 10 keyword bursts from three Chinese databases, the research results were categorized into two groups: resources and applications, with the latter still constituting the majority. The early study of the “fennel brain” might be attributed to its relevance to the medicinal quality of *I. verum* [36]. Subsequently, “Illicium” emerged as a research trend, potentially due to concerns regarding counterfeits in the resource pool of *I. verum*. In recent years, “biological activity” has become a prominent research trend, which might be associated with the antimicrobial and antioxidant characteristics of *I. verum* [4]. This shift in focus towards the biological activity of *I. verum* reflected the growing interest in understanding and harnessing the plant’s therapeutic potential.

## 4. Discussion

### 4.1. Resource of I. verum

The plants within the *Illicium* exhibit similar morphological characteristics, yet they can have significant differences in their chemical compositions. These differences have led to the emergence of various counterfeits of *I. verum* (Table 1). The *I. verum* itself is non-toxic and is utilized across multiple domains due to its content of volatile oils and trans-anethole, among other active constituents [2,8]. However, these morphologically similar counterfeits pose a threat to human health because they contain toxins [23]. Reports from Europe and the United States have indicated that accidental consumption of these counterfeits can lead to adverse neurological reactions [37]. The distribution and habitat preferences of different counterfeits may vary. For instance, *Illicium simonsii* Maxim is found in the mountainous regions of Yunnan, Guizhou, and Sichuan provinces in China [38], while *Illicium henryi* Diels is distributed in the moist areas of Hubei and Sichuan provinces, among other locations in China [9]. Although these counterfeits can be harmful if consumed directly, they possess significant value as they can be extracted to yield shikimic acid, a compound that has been used to treat avian influenza, among other applications [23]. Therefore, the judicious utilization of these *I. verum* counterfeits could be beneficial to humanity. Understanding the distribution of *I. verum* resources is crucial for the identification and differentiation of counterfeits.

The *I. verum* is native to China and Vietnam [23] and thrives in subtropical regions of southern Asia [9]. However, China stands out as the country with the richest resources and the capacity for substantial export [11]. The plant is predominantly found in provinces near the Tropic of Cancer in China, such as Guangxi, Guangdong, and Guizhou provinces in China, where it is cultivated extensively. Although *I. verum* has been introduced to other provinces, it has not reached a significant scale of cultivation [1]. Economic development has prompted the introduction of *I. verum* in many regions, but often, the outcomes have been less than satisfactory, failing to yield substantial economic benefits. There are several potential reasons for the poor performance of *I. verum* in introduced settings. Firstly, while introduction to colder climates may reduce the incidence of pests and diseases [39], different species vary in their adaptability, and excessively low temperatures can limit plant growth [40]. Secondly, when temperatures exceed the optimal range for the plant, net photosynthesis may decline due to increased respiration and stomatal closure [41], potentially preventing the plant from completing reproductive tasks and merely maintaining normal growth. Lastly, when plants are introduced to new environments, they may encounter new herbivores or re-encounter local herbivores [42], leading to a decrease in their adaptive capabilities. Biogenic volatiles are crucial substances that plants produce in response to biotic or abiotic stress, enhancing their survival [43]. Variations in the content and composition of volatiles in *I. verum* from different regions [2,4,23,44] indicate that different environments can influence the accumulation of active components in *I. verum*, which also limits the effectiveness of its introduction to new areas. Consequently, these factors contribute to the current distribution pattern of *I. verum*.

### 4.2. Hotspot and Trend of I. verum in Pharmaceutical Field

The fruits and foliage of *I. verum* contain a variety of important active compounds. These include over 20 active substances such as cis- and trans-anethole and tannins, a small number of nitrogenous components, 14 types of hydrocarbon constituents, and 22 oxygenated hydrocarbon derivatives [2,45,46]. These compounds are known for their antimicrobial, antioxidant, and insecticidal properties [47,48]. As a food-medicinal plant, the *I. verum* is widely used in both food and pharmaceutical applications. In countries such as China, Vietnam, and India, *I. verum* is frequently used in the preparation of meat dishes, while in Western countries, it is employed in the making of alcoholic beverages, teas, and jams [4]. Beyond enhancing the flavor of food, *I verum* is also utilized as a pharmaceutical agent due to its numerous active ingredients. For instance, it is used in the manufacturing of the antiviral drug oseltamivir (Tamiflu^®^) [49]. Trans-anethole and volatile oil are critical indicators of the medicinal quality of *I. verum*, and since the 2010 edition of the Chinese Pharmacopoeia, a minimum trans-anethole content of 4% has been stipulated [36]. An analysis of keyword clustering trends and hotspots in recent years reveals a pronounced focus on the pharmaceutical applications of *I. verum* (Figure 7 and Figure 8), highlighting the plant’s significance in medical research.

The primary research on *I. verum* has predominantly focused on the pharmaceutical domain due to several key reasons. Initially, advancements in extraction technologies have facilitated the identification of a growing number of active constituents. *I. verum*, like many traditional Chinese medicinal herbs, is a rich source of bioactive compounds [38]. The evolution of extraction techniques has led to the uncovering of an increasing variety of these compounds, thereby enhancing research efforts in their medicinal applications [50]. With the discovery of the harmful effects of antibiotic misuse in both livestock and human medicine, the search for alternatives to antibiotics has become a research priority [51,52]. The *I. verum*, with its antiviral and antioxidant properties, has emerged as a significant candidate in this context. Its potential to serve as a substitute for conventional antibiotics has garnered considerable attention in the scientific community. In conclusion, the future trajectory of research on *I. verum* is poised to continue its emphasis on pharmaceutical applications. The exploration of its bioactive components and the development of novel therapeutic strategies leveraging these properties will likely remain at the forefront of scientific inquiry.

## 5. Conclusions

China stands as the country with the richest resources of *I. verum*, predominantly centered around the Guangxi province and extending to surrounding provinces. Chinese scholars have the highest publication count in WOSCC and engage in close collaboration with researchers. The abundance of *I. verum* resources facilitates the advancement of research in academic institutions. Currently, the focus of research interest in *I. verum* is primarily within the medicinal domain, and the investigation of its bioactive properties is poised to become a new direction for future studies.

Due to the presence of many morphologically similar toxic plants within the same genus, the application of *I. verum* in the food industry has been constrained. However, *I. verum* and its related species possess significant medicinal value, as documented in references [4,5,6,7,8], which has led to a focused research interest in the medical field for *I. verum*. Based on the analysis of current research hotspots and trends regarding *I. verum* presented in this article, potential future developments in star anise research may include the following areas.

(1) The proportion of research into the selection and breeding of superior resources of *I. verum* and its counterfeits is increasing. Current trends in research from both China and internationally indicate a growing focus on antioxidant activity, which is emerging as a prevailing research trend. This outcome is expected to stimulate an increased demand for the antioxidant components of *I. verum* in the future. Consequently, the development of superior varieties of *I. verum* rich in antioxidant constituents or the cultivation of high-quality resources from related species within the same genus may present new avenues for research;
(2) The research proportion dedicated to the application of *I. verum* in the food sector is anticipated to expand. A growing number of researchers are likely to follow the prevailing trends in *I. verum* research, delving into the study of its chemical constituents. The discovery of an increasing array of compounds may facilitate the development of *I. verum* within the food industry, potentially leading to the creation of a variety of foods with distinctive flavors. The promotion of superior sources of *I. verum* is anticipated to augment the yield of this spice. Concurrently, an increase in production is likely to enhance the circulation of *I. verum* in the market, thereby elevating its proportion within the culinary sector.

## Figures and Tables

**Table 1:** Common adulterants and toxic parts of *I. verum*.

Species	Toxic of Location	Resources
*Illicium anisatum*	seeds and carpels	[22,27]
*Illicium floridanum*	fruits and leaves	[22,27]
*Illicium merrillianum*	pericarps	[22,27]
*Illicium majus*	fruits	[22,27]
*Illicium verum*	fruits	[22,27]
*Illicium arborescens*	fruits	[22,27]
*Illicium brevystylum*	fruits	[22,27]
*Illicium henryi*	fruits	[22,27]
*Illicium macranthum*	fruits	[22,27]
*Illicium majus*	fruits	[22,27]
*Illicium minwanense*	fruits	[22,27]
*Illicium simonsii*	fruits	[22,27]
*Illicium ternstroemioides*	fruits	[22,27]

**Table 2:** Top 10 Journals by publication count for *I. verum* research.

Type	Journal	Impact Factor	Categories	Number
WOSCC	Molecules	4.6	Biochemistry & molecular biology	11
	Industrial crops and products	5.9	Agricultural engineering	7
	Food chemistry	8.8	Chemistry, applied	5
	Journal of agricultural and food chemistry	6.1	Agriculture, multidisciplinary	5
	Poultry science	4.4	Agriculture, dairy, and animal science	5
	Food and chemical toxicology	4.3	Toxicology	4
	Phytotherapy research	7.2	Chemistry, medicinal	4
	Animal science journal	2.0	Agriculture, dairy, and animal science	3
	Environmental science and pollution research	5.8	Environmental sciences	3
	Evidence-based complementary and alternative medicine	2.65	Integrative and complementary medicine	3
CWV	China condiment	2.749	Light industrial handicrafts	60
	Food science and technology	1.623	Light industrial handicrafts	15
	Lishizhen medicine and materia medica research	1.496	Traditional Chinese medicine	14
	Science and technology of the food industry	2.592	Light industrial handicrafts	14
	Journal of Anhui Agricultural Sciences	0.900	Comprehensive agriculture	11
	Food science	3.894	Light industrial handicrafts	9
	Food research and development	2.251	Light industrial handicrafts	9
	Chinese journal of pharmaceutical analysis	1.665	Medicine	9
	Chinese traditional and herbal drugs	4.694	Traditional Chinese medicine	9
	Journal of the Chinese cereals and oils association	2.278	Light industrial handicrafts	11

Note: The impact factors and subject categories of international journals were obtained through the WOSCC. The impact factors listed were as of the year 2022, and where multiple subject categories are available, the one with the highest ranking in the Journal Citation Reports (JCR) was selected. Journals highlighted in red were those classified in the Q1 zone of the JCR. The acronym “CWV” represents a composite of the China National Knowledge Infrastructure (CNKI) (https://www.cnki.net/), Wanfang (https://w.wanfangdata.com.cn/index.html?index=true), and VIP (http://www.cqvip.com/). The impact factors and subject categories for journals were retrieved via the CNKI platform.

## Data Availability

No new data were created or analyzed in this study. Data sharing is not applicable to this article.

## Supplementary Materials

The following supporting information can be downloaded at https://www.mdpi.com/article/10.3390/foods13101510/s1, Figure S1: Morphology of spring and autumn fruits of *I. verum*. Figure S2: Institution collaboration network in *I. verum* based on three Chinese databases. Figure S3: Researcher collaboration network in *I. verum* based on three Chinese databases. Figure S4: Visual mapping of keyword in *I. verum* using CiteSpace based on three Chinese databases. Figure S5: The keyword time zones of *I. verum* based on three Chinese databases.
