# Supplementary material for: Based on CiteSpace Insights into Illicium verum Hook. f. Current Hotspots and Emerging Trends and China Resources Distribution
Source: Foods. 2024 May 13;13(10):1510. doi: 10.3390/foods13101510 (PMC11119909; doi:10.3390/foods13101510)
Supplement: Supplementary file 1 [file foods-13-01510-s001.zip › foods-2976625-supplementary.pdf]

Figure S1

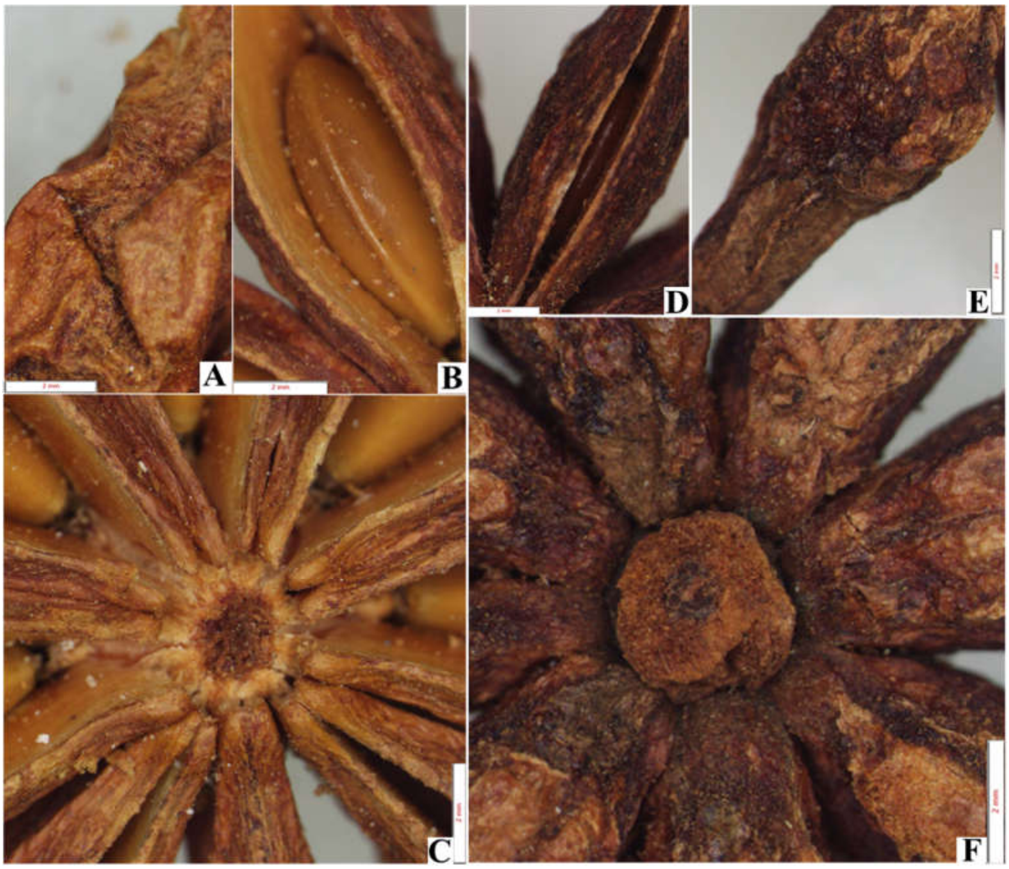

Figure S1. Morphology of spring and autumn fruits of *I. verum*. A: The peel morphology of the autumn fruit of *I. verum*; B: The morphology of fruit cracks in the autumn fruit of *I. verum*; C: The shape of the fruit plate of the autumn fruit of *I. verum*; D: The fruit crack morphology of *I. verum* spring fruit; E: The peel morphology of *I. verum* spring fruit; F: The Fruit Plate Morphology of *I. verum* Spring Fruit

Figure S2

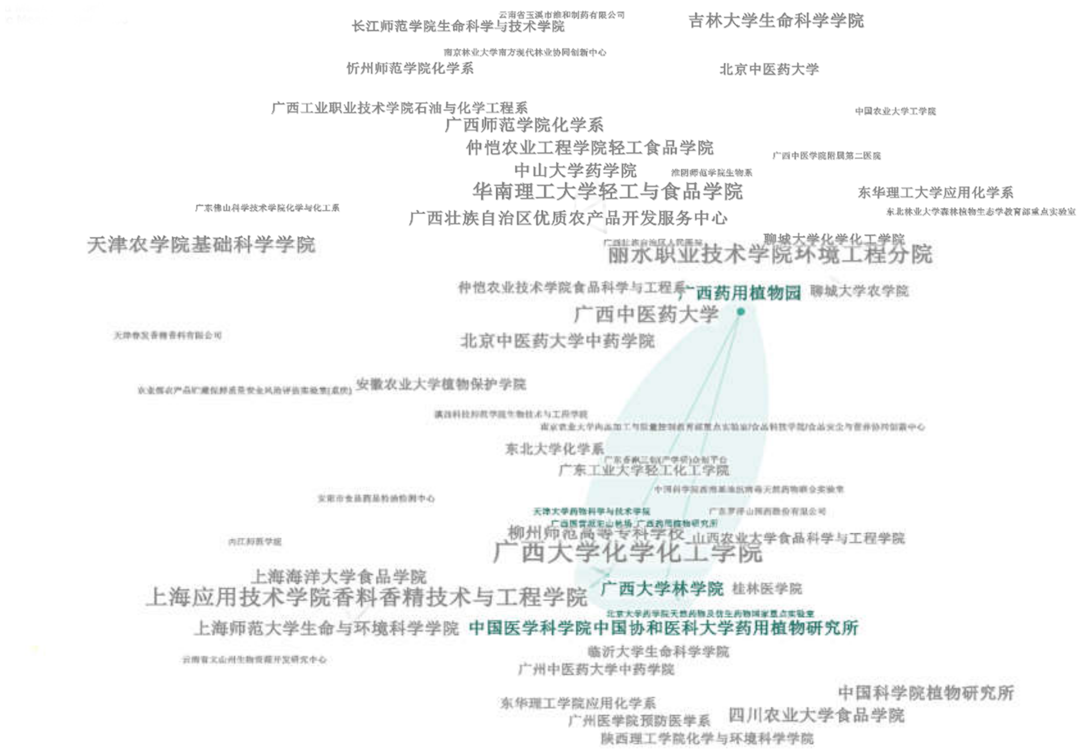

Figure S2. Institution collaboration network in *I. verum* based on three Chinese databases. The largest font in the figure is "School of Chemistry and Chemical Engineering, Guangxi University", indicating that this institution has the largest number of studies on star anise. Most of the research institutes in the light green module are located in Guangxi Zhuang Autonomous Prefecture

Figure S3

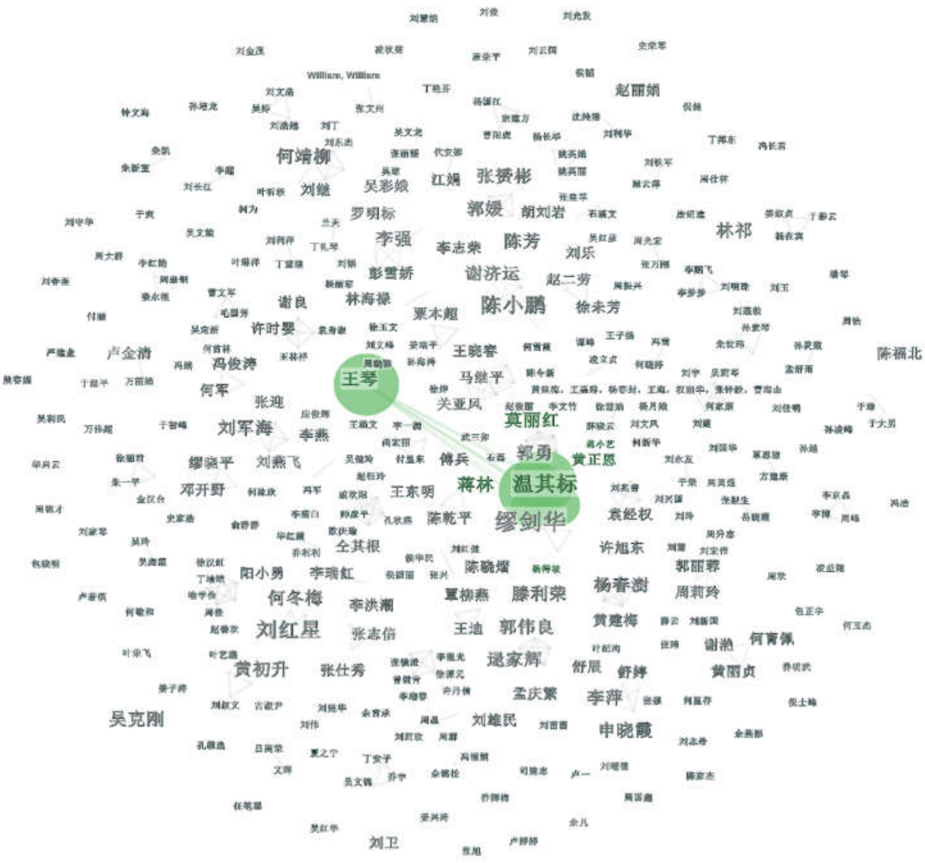

Figure S3. Researcher collaboration network in *I. verum* based on three Chinese databases. The green modules represent exchanges between Chinese researchers who publish the most star anise.

Figure S4

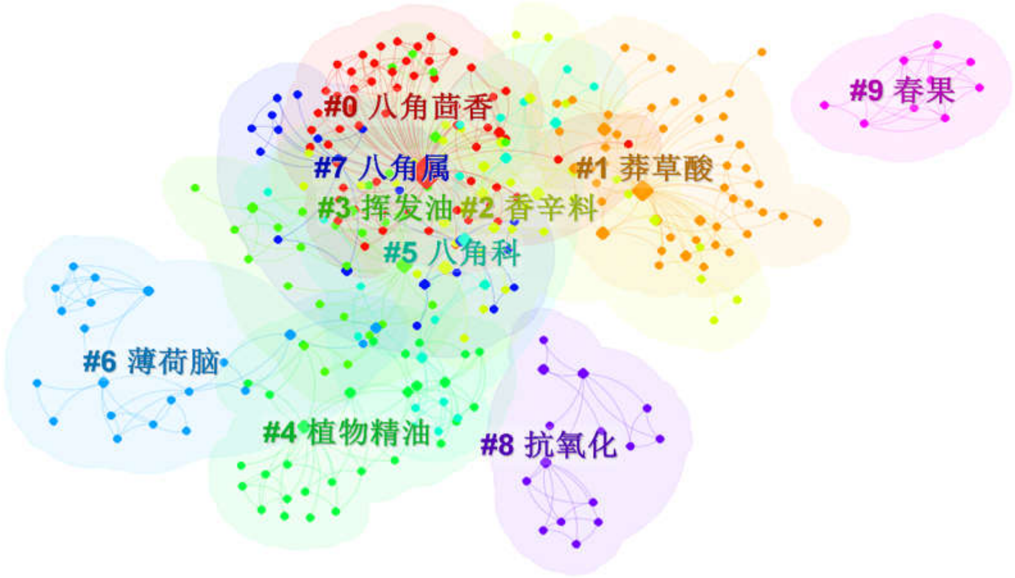

Figure S4. Visual mapping of keyword in *I. verum* using CiteSpace based on three Chinese databases. The keyword clustering of *I. verum* in three Chinese databases could likewise be divided into two main categories: resources and application of *I. verum*.

Figure S5

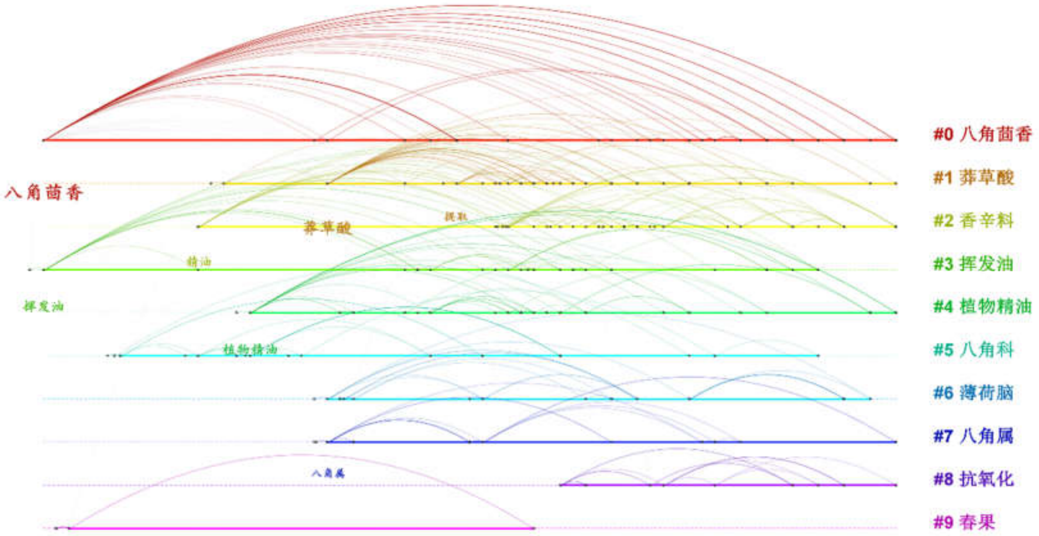

Figure S5. The keyword time zones of *I. verum* based on three Chinese databases. The keyword clustering of *I. verum* in three Chinese databases could likewise be divided into two main categories: resources and application of *I. verum*. The resource research of *I. verum* is the earliest.
